# There Goes the Neighbourhood—A Multi‐City Study Reveals Ticks and Tick‐Borne Pathogens Commonly Occupy Urban Green Spaces

**DOI:** 10.1111/zph.13208

**Published:** 2025-01-16

**Authors:** Jani J. Sormunen, Satu Kylänpää, Ella Sippola, Riikka Elo, Nosheen Kiran, Veli‐Matti Pakanen, Eva R. Kallio, Eero J. Vesterinen, Tero Klemola

**Affiliations:** ^1^ Department of Biology University of Turku Turku Finland; ^2^ Biodiversity Unit University of Turku Turku Finland; ^3^ Turku Bioscience Centre University of Turku and Åbo Akademi University Turku Finland; ^4^ Finnish Museum of Natural History (LUOMUS) University of Helsinki Helsinki Finland; ^5^ Tampere Museum of Natural History, Museum Center Vapriikki Tampere Finland; ^6^ Department of Biological and Environmental Science University of Jyväskylä Jyväskylä Finland; ^7^ Ecology and Genetics Research Unit University of Oulu Oulu Finland

**Keywords:** landscape features, public health, risk index, tick‐borne pathogens, ticks, urban green spaces

## Abstract

**Introduction:**

Humans acquire tick‐borne pathogens (TBPs) from infected ticks contacted during outdoor activities. Outdoor activity is at its highest in urban green spaces, where the presence of tick populations has increasingly been observed. Consequently, more insight into factors influencing the presence of ticks therein is needed. Here, we assess the occurrence of ticks and several TBPs in urban green spaces in Finland, estimate related human hazard and assess how landscape features influence tick and TBP occurrence therein.

**Methods:**

Ticks collected from five cities during 2019–2020 were utilised. *Borrelia*, *Rickettsia*, *Neoehrlichia mikurensis*, *Anaplasma phagocytophilum* , *Babesia* and TBEV were screened from ticks using qPCR. Various landscape features were calculated and utilised in generalised linear mixed models to assess their contribution towards tick and TBP occurrence in green spaces. Finally, human population density proximate to each study site was calculated and used to create population‐weighted risk indices.

**Results:**

*Borrelia* were the most common pathogens detected, with 22% of nymphs and 43% of adults infected. Increasing forest cover had a positive effect on the densities of nymphs and adults, whereas forest size had a negative effect. Middling percentages of artificial surfaces predicted higher nymph densities than low or high values. Human population‐weighted risk estimates were highly varied, even within cities. A positive correlation was observed between total city population and risk indices.

**Conclusions:**

Ticks and TBPs are commonplace in urban green spaces in Finland. Enzootic cycles for *Borrelia* and *Rickettsia* appear to be well maintained within cities, leading to widespread risk of infection therein. Our results suggest that nymph densities are highest in urban forests of medium size, whereas small or large forests show reduced densities. Green spaces of roughly similar risk can be found in cities of different sizes, emphasising that the identification of areas of particularly high hazard is important for effective mitigation actions.

**Summary**

- Tick field collection and pathogen analysis data from urban green spaces in five Finnish cities was combined, revealing that tick and tick‐borne pathogen populations are common and well maintained therein.
- Increasing percentage of forest cover in urban green spaces predicted higher densities of ticks, whereas increasing size of the forest had a negative impact. Likewise, tick densities appeared highest when artificial surfaces covered roughly half of the area adjacent to green spaces.
- Human population densities proximate to each green space were estimated, and population density–weighted risk indices calculated, revealing that green spaces in cities of different sizes may present equal hazards.

## 1 Introduction

Ticks and tick‐borne diseases (TBDs) are an acknowledged and growing threat to human health, with several hundred thousand cases of TBDs reported annually worldwide (Marques, Strle, and Wormser 2021). Lyme borreliosis, caused by *Borrelia burgdorferi* sensu lato (s.l.) spirochetes, is the most common TBD in the Northern Hemisphere, with dramatic increases in disease burden observed over the past few decades (Stark et al. 2023). European ticks also harbour several other medically important pathogens, such as the tick‐borne encephalitis virus (TBEV), bacterial pathogens *Neoehrlichia mikurensis*, *Rickettsia* spp. and *Anaplasma phagocytophilum* and protozoan parasites *Babesia* spp. Regardless of the specific pathogen in question, humans acquire pathogens causing TBDs from the bites of infected ticks (Acari: Ixodidae). As such, the relative risk of acquiring a TBD has typically been measured with an entomologic hazard index depicting how many infected ticks are found in a given area, typically the density of infected nymphs (DIN) (Mather et al. 1996). However, the likelihood of TBDs is also dependent on human exposure to infected ticks. Human TBD rates are likely thus influenced not only by the presence of pathogen‐carrying ticks but also by human activity. Human outdoor activity may be expected to be at its highest in green spaces in cities. Therefore, urban ticks may form a particularly important source of TBDs, even if infected ticks are present in lower densities than in more sylvatic habitats (Bourdin et al. 2023). Consequently, identifying the factors that determine the presence and abundance of ticks and tick‐borne pathogens (TBPs) in urban green spaces is important from a public health perspective (Rizzoli et al. 2014; Hansford et al. 2022).

To incorporate human presence into the risk measurements, the use of visitor count and population density data have been attempted (Sormunen, Kulha, et al. 2020; Janzén et al. 2024). Likewise, the use of mobile device data in activity assessments has been explored, but the methodology has not yet been implemented for tick risk assessments (Monz et al. 2019). Visitor count data are likely accurate in depicting human visits to green spaces but typically available only for limited areas. However, a recent study conducted in urban green spaces in Stockholm, Sweden, observed a high correlation between camera‐based visitor counts to urban green spaces and census‐based population estimates of areas near the green spaces (Janzén et al. 2024). Consequently, adjacent population density is expected to predict visitor counts to nearby urban green spaces, enabling the use of more commonly available population density data for comprehensive and geographically extensive risk assessments incorporating human activity (Sormunen, Kulha, et al. 2020; Janzén et al. 2024; Cayol et al. 2018).

Although ticks can be found in many different types of urban green areas along with TBPs capable of infecting humans (Rizzoli et al. 2014; Hansford et al. 2022), it has been observed that tick densities often decrease with increasing proportion of artificial surfaces, indicating suboptimal conditions for tick population upkeep near city centres (Buczek et al. 2014; Hauck, Springer, et al. 2020). However, this trend is not uniform, as in some cases urban green spaces near city centres may even have higher tick densities than surrounding areas (Hansford et al. 2022; Cayol et al. 2018; Borşan et al. 2020). This variation across studied cities is likely influenced by a plethora of factors related to, for instance, city structure (e.g., green corridors; Heylen et al. 2019) and geographical location. To account for this variation, the determination of factors influencing tick and TBP occurrence in different cities is important. However, it is equally paramount not to focus only on individual cities, but to incorporate several cities in analyses to determine whether any widely applicable predictors exist.

Relatively few studies of ticks in urban or suburban areas in Finland have been conducted, but they have revealed that ticks and TBPs are present also in urban green spaces (Sormunen, Kulha, et al. 2020; Cayol et al. 2018, 2017; Klemola et al. 2019; Pakanen et al. 2020; Zakham et al. 2021; Junttila et al. 1999; Mäkinen et al. 2003). There has even been some indication of certain TBPs being more common in urban than in less urbanised areas locally (Sormunen, Kulha, et al. 2020; Sormunen et al. 2016; Cayol et al. 2018; Klemola et al. 2019). Likewise, previously conducted (Laaksonen et al. 2017) and ongoing (www.punkkilive.fi/en) crowdsourcing studies have revealed that tick contacts are commonly reported from within cities in Finland. However, crowdsourced ticks removed from humans, dogs or cats within cities may have been acquired from elsewhere, so there is an element of uncertainty in these observations. Likewise, the number of citizen science observations is inherently linked to population density, making assessments of tick abundance challenging (Sormunen et al. 2023). Consequently, to more accurately assess the occurrence patterns of ticks and TPBs in urban green spaces, field studies are required.

In the current study, we assess the occurrence of ticks and several TBPs in urban green spaces in five cities in Finland, to quantify potential human infection risks in these areas. We estimate the human populationweighted risk indices regarding *Borrelia* infection to determine if risks are equal across cities of different sizes. In addition, we use these data to determine whether uniform trends in landscape structure influencing tick or TBP presence can be identified across the cities.

## 2 Materials and Methods

### 2.1 Tick Data

**FIGURE 1:**
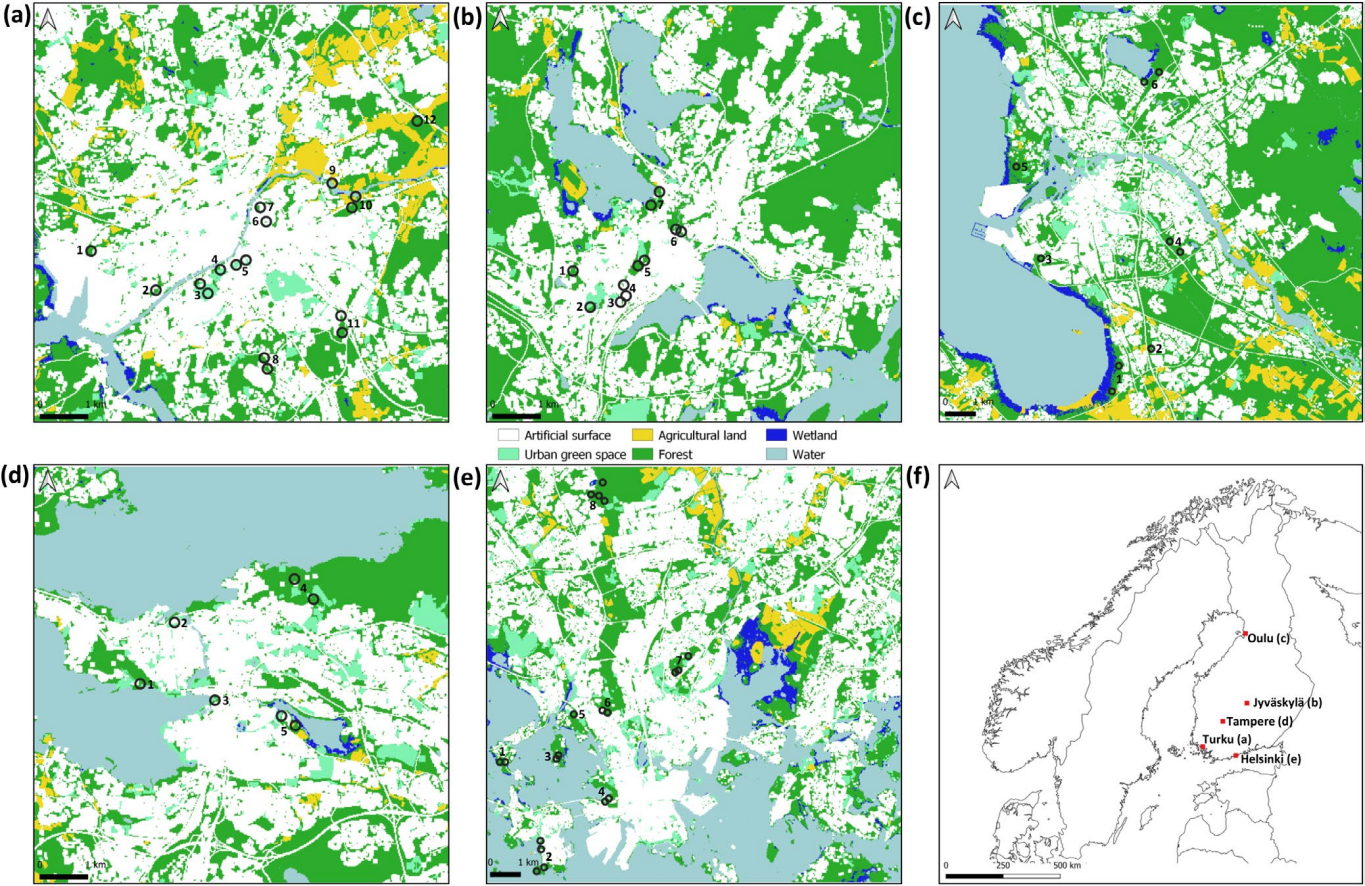
Locations of study sites within Turku (a), Jyväskylä (b), Oulu (c), Tampere (d) and Helsinki (e), and locations of the cities in Finland (f). Maps (a)–(e) are Corine Land Cover maps (CLC2018, 20 m resolution; Finnish Environment Institute), visualised according to the first level CLC2018, with the exception of urban green space (Level 3, Classes 141 and 142). Note that one study site can be represented by more than one circle, as described in Section 2. Location names are listed in Table S1.

Ticks were collected by cloth dragging in 2019 from the city of Turku (human population ~198,000) and in 2020 from the cities of Tampere (~249,000), Jyväskylä (~146,000) and Oulu (~212,000) (Figure 1). Likewise, previously published tick abundance and TBP data from the capital city, Helsinki (population ~665,000), was included in all analyses (Sormunen, Kulha, et al. 2020). All the study cities are among the seven largest cities in Finland. Dragging was conducted once per month from May to September in 11 (Turku), eight (Helsinki), five (Tampere), seven (Jyväskylä) or six (Oulu) study sites located in urban green spaces within or in close proximity to the city centres (Figure 1). In Tampere, Jyväskylä and Oulu, 500 m were dragged at each study site each month, in 10‐m sections. In Turku, the monthly distance dragged varied between 400 and 1000 m, with some variation across sites (Table S1). Specific dragging data for Helsinki has previously been reported (Sormunen, Kulha, et al. 2020). Ticks were collected from the cloths with tweezers and placed in Eppendorf tubes containing 70% ethanol. Collected ticks were sent by mail to the University of Turku for further processing and morphological identification using stereo microscopes (Estrada‐Peña, Mihalca, and Petney 2018).

### 2.2 Laboratory Analyses

Total DNA and RNA were extracted from adult and nymphal ticks using NucleoSpin RNA kits and RNA/DNA buffer sets (Macherey‐Nagel, Germany), following the kit protocols (NucleoSpin 96 RNA Core Kit: Rev. 05/April 2014 and RNA/DNA buffer set: Rev. 09/April 2017). RNA extracts were stored at −80°C for later analyses. DNA extracts were stored at −20°C.

DNA samples were screened for bacterial pathogens *Borrelia* spp., *A. phagocytophilum* , *N. mikurensis* and *Rickettsia* spp., as well as protozoan parasites *Babesia* spp. TBEV was screened from tick RNA samples from Turku but not from other cities, due to financial restraints of the project. For a portion of the samples, molecular species identification ( *Ixodes ricinus* or *Ixodes persulcatus* ) was carried out using an *ITS2*–targeted qPCR assay, as previously described (Sormunen et al. 2016). The primers ‘Bb23S’ (Courtney et al. 2004), originally designed for screening *B. burgdorferi* s.l., have been observed to also amplify *Borrelia miyamotoi* , a relatively rare relapsing fever spirochete not part of the *B. burgdorferi* s.l. species complex (unpublished own data). As such, samples that were not screened for specific *Borrelia* species have to be considered as carrying *Borrelia* spp. rather than *B. burgdorferi* s.l. Consequently, we refer to *Borrelia* spp. when collectively discussing all detections of *Borrelia* made in the current study.

Analyses regarding *Borrelia* were carried out on individual DNA samples. For the screening of all other pathogens, samples were first analysed in pools (12 samples per pool, 5 μL of each sample added to the pool) due to low expected prevalence. Individual samples from a pool found positive were subsequently reanalysed separately. The primers used for each pathogen are reported in Table S2. Real‐time quantitative PCR assays were carried out using SensiFAST Probe Lo‐ROX Kit (for DNA) and SensiFAST Probe Lo‐ROX One‐Step Kit (for RNA) (Bioline, Germany). All DNA/RNA samples were analysed in two replicate reactions carried out on 96‐ or 384‐well plates. At least two blank water samples were used as negative controls in each assay. Samples were considered positive when successful amplification was detected in both replicate reactions or in two consecutive assays. See Appendix S1 for thermal cycling profiles, information on positive controls and mastermix contents (Table S3).

### 2.3 Environmental Variables

We quantified the proportions of different main (Level 1) CORINE Land Cover classes within a 500‐m buffer around study sites (CLC2018, 20 m resolution; Finnish Environment Institute). For some study sites, several buffers were included to cover the whole collection area. In such cases, averages of the proportions of land use classes were used in analyses. In addition to the Level 1 classes, we separated ‘green urban areas’ (Class 141) and ‘sport and leisure facilities’ (Class 142) as areas suitable for tick inhabitation from other artificial surfaces, and added them as a separate new Level 1 main class (‘urban green space’). We also calculated an *urbanisation index* for study sites, following Janzén et al. (2024). Briefly, this is a metric that subtracts open water from other land use classes, subsequently providing a measure of how much (%) of the land area artificial surfaces cover. The values range from 0 (completely natural or seminatural area) to 100 (only artificial surfaces) (Janzén et al. 2024).

To incorporate a measure of environmental heterogeneity into the analyses, we calculated the densities of different edge parameters (edge in m/ha) between the CLC land use classes within the 500‐m buffer areas. We calculated edges between different land use classes (‘LandUseEdge’), suitable habitats for ticks (‘HabitatEdge’; classes other than open water or artificial surface) and edges between artificial surfaces and other land use classes (‘ArtificialEdge’). Furthermore, to include an estimate of forest and suitable tick habitat size at study sites, we calculated the largest uniform forest/suitable tick habitat (CLC classes excluding artificial surfaces and open water) at study sites from the CLC data. Because of the high variation between values, we did a Z‐standardisation for values of forest/suitable habitat size. Finally, we calculated the total human population density within 500 m and 1 km from the study sites, utilising population census data (Statistics Finland; population densities in 1 km^2^ grids). All geographical data manipulation and analyses were made with QGIS Version 3.28.

### 2.4 Population Density–Weighted Infection Risk

We calculated a human population density–weighted infection risk index regarding *Borrelia* within 500 and 1000 m of each study site (Sormunen, Kulha, et al. 2020). Only *Borrelia* were used for risk analyses, as local species of *Rickettsia* do not cause human diseases and insufficient numbers of positive ticks were found for other pathogens (Table 1). Five hundred metres has been suggested as the typical range for daily outdoor activities of local residents (‘neighbourhood’ in Fischhoff et al. 2019). However, we also added estimates from a longer range (1000 m), as green spaces within cities are surrounded by highly built‐up areas, potentially leading to longer distances to recreational areas. Weighted infection risk was calculated by multiplying local estimates of densities of infected ticks (DIN/DIA; tick density/100 m^2^ × proportion of ticks carrying *Borrelia*; nymphs for *I. ricinus* , adults for *I. persulcatus* ) with estimates of local human population density.

**TABLE 1:** City‐ and tick life stage (nymph or adult)‐specific numbers of samples screened and positive for various tick‐borne pathogens.

City	Analysed nymphs	Samples positive for pathogen (prevalence ± 95% confidence interval)				Analysed adults	Samples positive for pathogen (prevalence ± 95% confidence interval)			
		*Borrelia* spp.	*Rickettsia* spp.	*Babesia* spp.	*Anaplasma phagocytophilum*		*Borrelia* spp.	*Rickettsia* spp.	*Babesia* spp.	*Anaplasma phagocytophilum*
Turku	372	97 (26% ± 5%)	57 (15% ± 4%)	1 (0.3% ± 0.5%)	0	47	18 (38% ± 14%)	8 (17% ± 11%)	0	0
Jyväskylä	370	85 (23% ± 4%)	31 (8% ± 3%)	0	2 (1% ± 1%)	10	1 (10% ± 20%)	1 (10% ± 20%)	0	0
Tampere	353	40 (11% ± 3%)	21 (6% ± 3%)	0	1 (0.3% ± 1%)	27	3 (11% ± 0.12)	4 (15% ± 13%)	0	0
Oulu	68	20 (29% ± 11%)	3 (4% ± 5%)	0	0	227	111 (49% ± 7%)	4 (2% ± 2%)	0	0
Helsinki^a^	1386	319 (23% ± 2%)	109 (8% ± 1%)	5 (0.4% ± 0.3%)	15 (1% ± 1%)	—	—	—	—	—
Total	2549	561 (22% ± 2%)	221 (9% ± 1%)	6 (0.2% ± 0.2%)	18 (0.7% ± 0.3)	311	133 (43% ± 6%)	17 (6% ± 3%)	0	0

^a^ Data from Sormunen, Kulha, et al. (2020).

### 2.5 Statistical Analyses

We aimed to identify landscape features that are associated with the density of ticks and the pathogen prevalence in ticks in urban green spaces across the several cities. We grouped all cities inhabited by *I. ricinus* (Helsinki, Turku, Tampere and Jyväskylä) for analysis. As Oulu was the only *I. persulcatus* city, with only six study sites, no meaningful analyses regarding environmental factors could be conducted for *I. persulcatus* . In all analyses concerning tick densities, the response variable was the life stage–specific tick density/100 m^2^, separately for each study site and month. For pathogen analyses, we used sample‐specific pathogen occurrence data (binary data).

More specifically, we wanted to see how the presence of suitable tick habitats and, contrastingly, the level of anthropogenic influence affect tick densities and the prevalence of *Borrelia* and *Rickettsia* (only pathogens with sufficient positive samples for analyses) in urban green spaces. We ran separate models for both questions to be able to discern differences in responses to highly correlated and contrasting variables (e.g., proportions of forests and artificial surfaces). We checked correlation of each explanatory variable and removed highly correlated (*r* > 0.7) variables based on expert assessment of variable relevance. After removal of correlated variables, analyses regarding suitable tick habitats included the proportion of forest at study sites, edges between all land use classes and all suitable habitats (‘LandUseEdge’ and ‘HabitatEdge’) and the size of forest adjacent to each study site as explanatory variables. To study the effects of the level of anthropogenic influence, we included the urbanisation index (Janzén et al. 2024), the quadratic polynomial of urbanisation index (urbanisation index^2^), the edge between artificial surfaces and other land use classes (‘ArtificialEdge’) and population density as explanatory variables.

We ran models for tick densities using generalised linear mixed models (GLMMs) with a negative binomial distribution. Log‐transformed monthly cloth dragging distance [log(distance_dragged)/100] at each study site was used as an offset term in models. Study site and month were included as random factors to account for spatial and temporal variation in data. Models for *Borrelia* and *Rickettsia* were similar to those of tick densities, but with binary distribution (modelling for ‘1’), no offset term and study site as the sole random factor.

Finally, we analysed differences between tick densities across cities using generalised linear models (GLMs), with negative binomial distributions and log link functions (separate models for different life stages). Log‐transformed distance of cloth dragging at each study site was used as an offset term.

All the GLMs were run with the GLIMMIX procedures of SAS v.9.4, using maximum or residual pseudo‐likelihood estimation. We checked for potential overdispersion in the models by comparing the variance of the Pearson residuals to 1, but no overdispersion was observed (values < 1).

## 3 Results

### 3.1 Tick Species and Abundance

**FIGURE 2:**
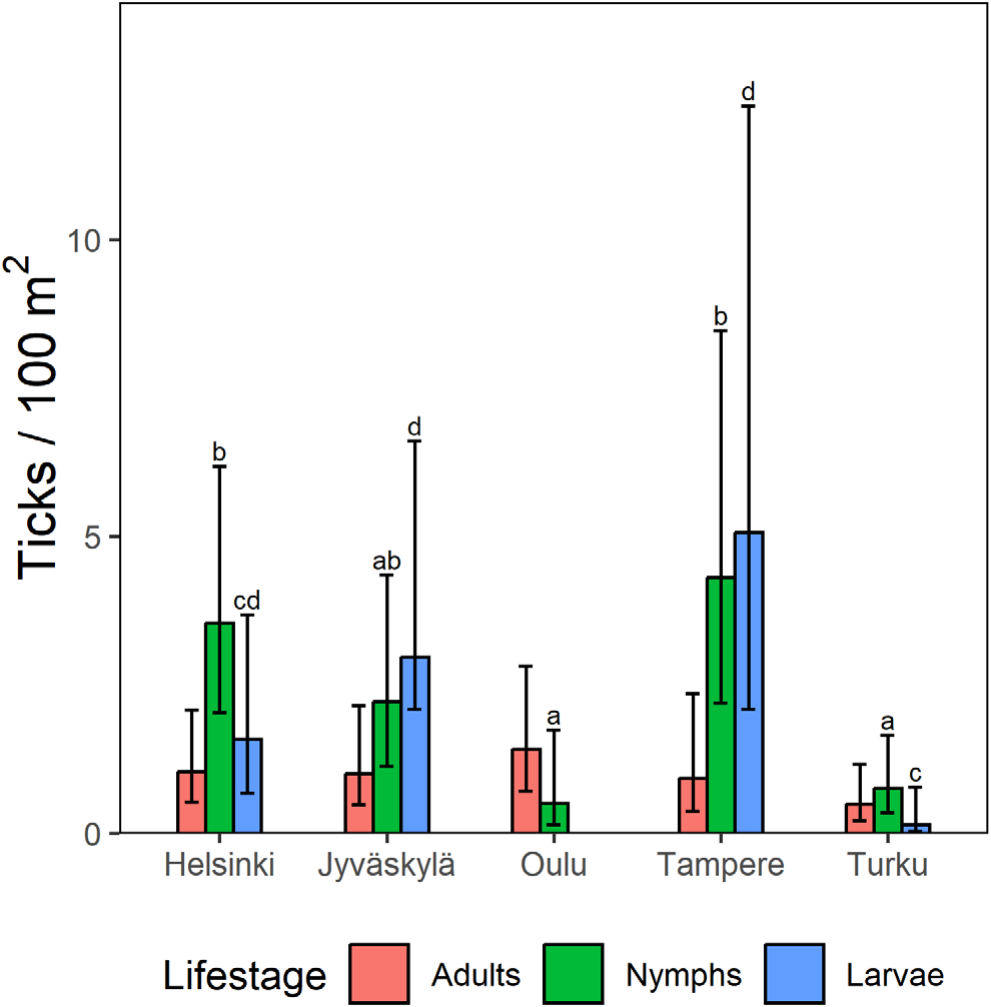
Densities of *Ixodes ricinus* and *Ixodes persulcatus* in different study cities in Finland, with 95% confidence limits. No differences between cities were observed for adult densities, whereas for nymphs and larvae, differences between cities with different letters are statistically significant in pairwise comparisons (*p* < 0.05).

A total of 1282 adult ticks, 4727 nymphs and 3529 larvae were collected from Turku, Helsinki, Tampere and Jyväskylä during roughly 143 km of cloth dragging (Table S1; Sormunen, Kulha, et al. 2020). Based on previously published data (Klemola et al. 2019; Cayol et al. 2017), the morphological identification of 50 adults from each city and molecular species identification for 350 (Tampere) and 548 (Turku) nymphs, the species present in these cities was determined to be *I. ricinus* . Only from Tampere, one adult male caught in May from study site Kauppi was identified as *I. persulcatus* . From Helsinki, only *I. ricinus* have been identified (Sormunen, Kulha, et al. 2020). Furthermore, 229 adults and 78 nymphs were collected from Oulu during 15 km of cloth dragging (Table S1). Based on previously published data (Pakanen et al. 2020) and the molecular identification of 203 adults and 28 nymphs, the species present in Oulu was determined to be *I. persulcatus* . No tick larvae were found in Oulu. City‐ and month‐specific numbers and cloth dragging lengths are reported in Table S1. Spatial heterogeneity in tick densities was high both between and within cities (Figure 2 and Table S1). Average densities varied between 0.49 and 1.42 ticks/100 m^2^ for adults, 0.5 and 4.31 for nymphs and 0 and 5.1 for larvae, depending on the city (Figure 2).

**FIGURE 3:**
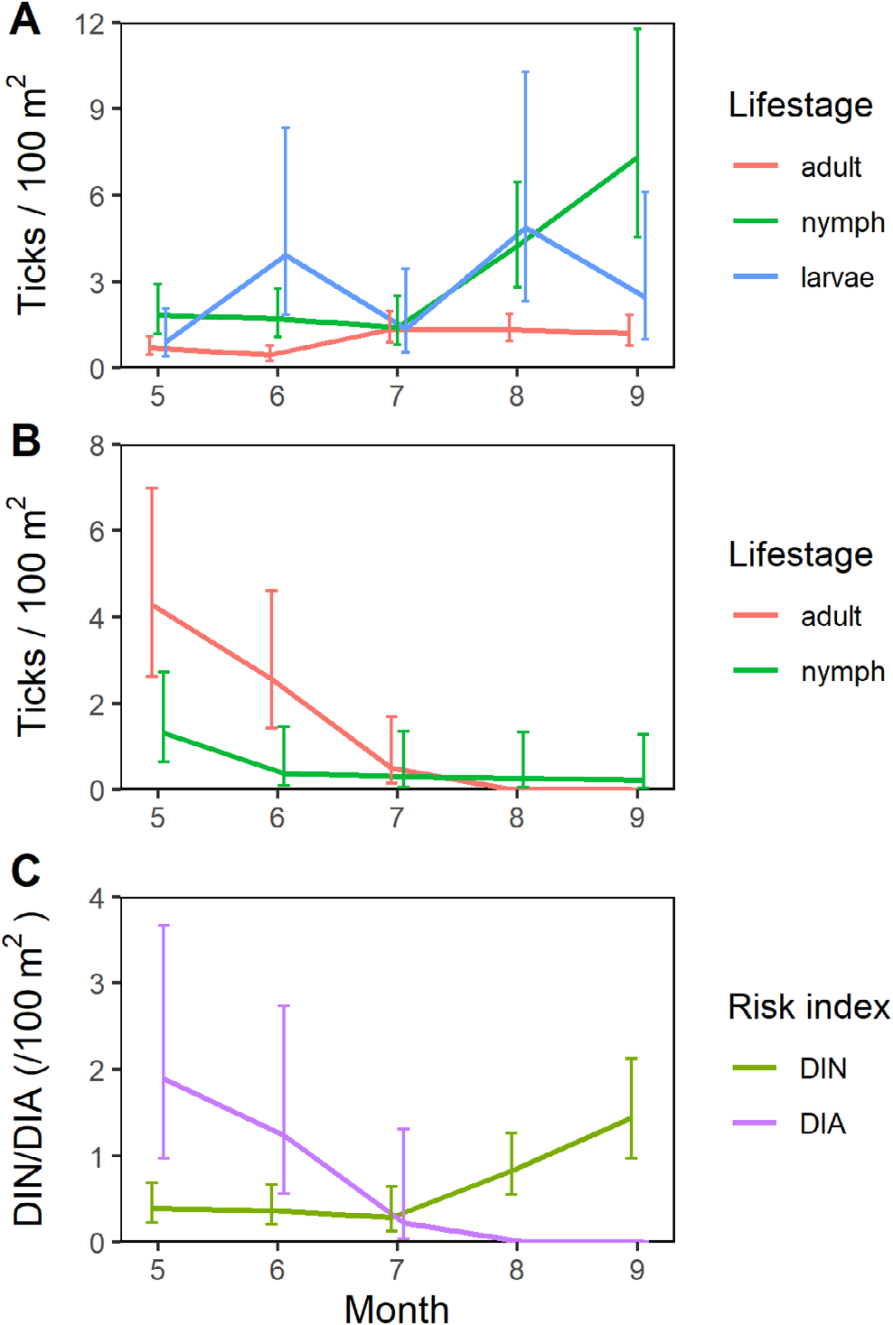
Seasonal activity patterns of *Ixodes ricinus* (A) and *Ixodes persulcatus* (B), and density of infected nymphs or adults (C), with 95% confidence limits. In *I. ricinus* cities, risk index is calculated as the density of nymphs infected with *Borrelia* spp. (DIN), whereas in *I. persulcatus* cities, the density of infected adults (DIA) is used.

For *I. ricinus* , the activity of all life stages appeared bimodal or unimodal, with the lowest values typically in June/July and the highest values in August/September (Figure 3A). The activity of *I. persulcatus* was unimodal, with activity mostly in May and June (Figure 3B). Monthly DIN or DIA followed patterns of tick activity. In cities inhabited by *I. ricinus* , the highest DIN was observed in August and September, but infected ticks were present from May to September (Figure 3C). For *I. persulcatus* , DIA was highest in May, and infected ticks were observed only from May to July.

### 3.2 Tick‐Borne Pathogens

A total of 1163 nymphs and 311 adults were analysed for the listed pathogens (Table 1). Observing all five study cities together, *Borrelia* spp. were the most common pathogens detected from urban study sites (found in 33/37 sites), with prevalence of 22% ± 2% (presented with 95% confidence limits) for nymphs and 43% ± 6% for adults. Other pathogens were less common, with *Rickettsia* spp. being the second most prevalent with 9% ± 1% of nymphs and 6% ± 3% of adults carrying the pathogens in urban study sites (Table 1). *Rickettsia* spp. were found in 32/37 study sites. Other screened pathogens were rare (Table 1). *N. mikurensis* was detected from only one nymph from Tampere (1/353; samples from Jyväskylä were not analysed for *N. mikurensis*). No nymphs carrying TBEV were detected from Turku.

### 3.3 Influence of Environmental Variables on Tick Densities

**FIGURE 4:**
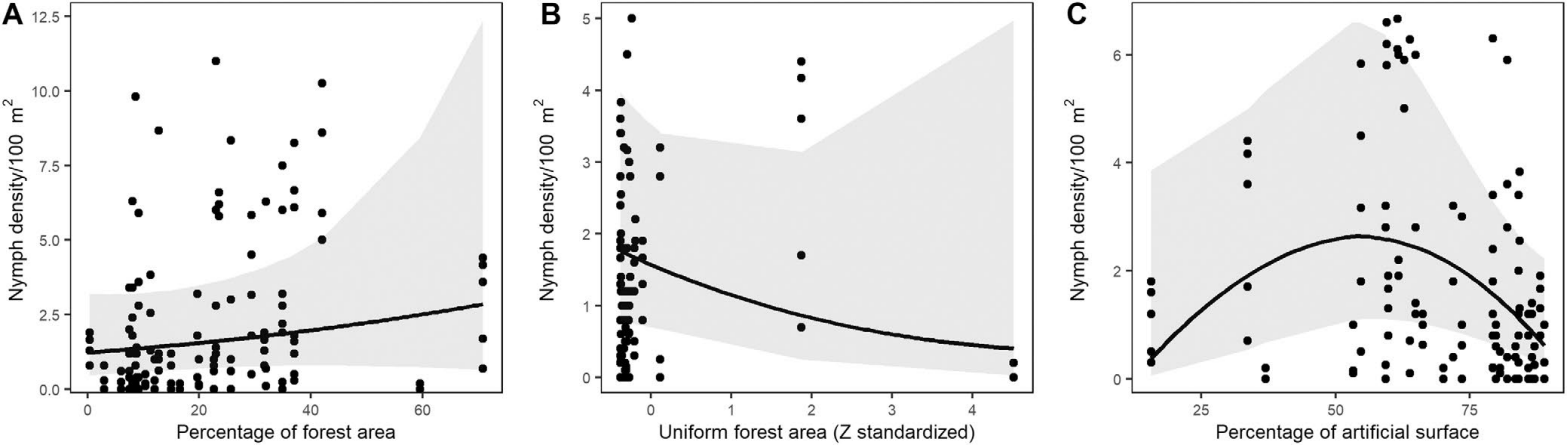
Observed nymph densities (dots) and predictions from the statistical models (lines with 95% confidence limits), presented for: percentage of forest cover (A), size of forest area (B) and percentage of artificial surface (C). Estimates were obtained from the GLMM models encompassing all *I. ricinus* cities. Only predictions from statistically significant models (*p* < 0.05) are presented.

Increasing proportion of forest was observed to have a positive effect on the densities of *I. ricinus* nymphs and adults (Table 2 and Figure 4A). Contrastingly, increasing forest size was observed to have a negative effect on the densities of both nymphs and adults (Table 2 and Figure 4B). Likewise, we observed a significant negative effect of the quadratic polynomial of the urbanisation index regarding nymph densities, indicating that low or high proportions of artificial surfaces predicted lower nymph densities than middling values (Figure 4C). No significant predictors were observed for *Borrelia* or *Rickettsia*. Likewise, likely due to high variation in numbers, many zero observations, and the inclusion of two random factors, models for larvae failed to converge.

**TABLE 2:** Results of generalised linear mixed models for *Ixodes ricinus* nymph (A and B) and adult (C) densities. Parameter estimates (in log scale and with 95% confidence limits) are given in the column ‘Estimate’. Predictions of the nymph models are depicted graphically (Figure 4). Significant variables are bolded.

Fixed effects	Ndf	Ddf	*F*	*p*	Estimate
(A) Nymphs and suitable urban habitats					
**Forest cover**	**1**	**28.9**	**7.05**	**0.01**	**4.7 (1.1 to 8.4)**
**Uniform forest area**	**1**	**80.7**	**6.19**	**0.01**	**−1.1 (−2 to −0.2)**
‘LandUseEdge’	1	27.44	0.09	0.22	−0.3 (−0.8 to 0.2)
‘HabitatEdge’	1	24.9	1.60	0.76	−0.06 (−0.5 to 0.3)
(B) Nymphs and anthropogenic influence					
Urbanisation index	1	22.7	0.00	0.96	0.03 (−1.3 to 1.4)
**Urbanisation index** ^**2**^	**1**	**21.6**	**5.35**	**0.03**	**−1.6 (−3.1 to −0.2)**
‘ArtificialEdge’	1	20.7	0.31	0.58	−0.14 (−0.6 to 0.4)
Population	1	21.8	1.53	0.23	4 × 10^−5^ [(−2 to 0.9) × 10^−5^]
(C) Adults and suitable urban habitats					
**Forest cover**	**1**	**26.4**	**6.31**	**0.02**	**3.6 (0.7 to 6.5)**
**Uniform forest area**	**1**	**55.0**	**4.74**	**0.03**	**−1.05 (−2 to −0.08)**
‘LandUseEdge’	1	27.9	0.60	0.44	−0.13 (−0.5 to 0.2)
‘HabitatEdge’	1	26.0	0.37	0.56	−0.09 (−0.4 to 0.2)

### 3.4 Population‐Weighted Infection Risk

**FIGURE 5:**
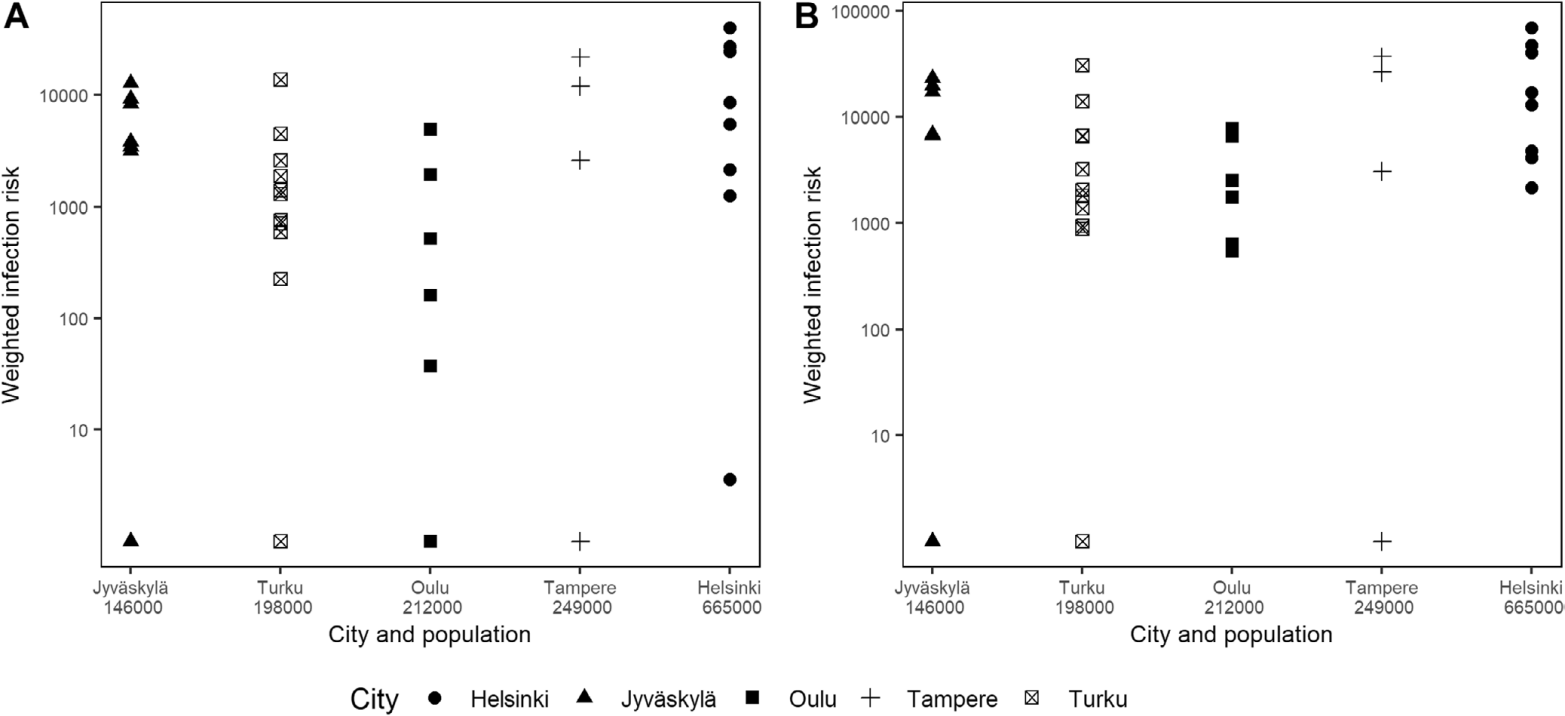
Population density–weighted infection risk estimates calculated within: (A) 500 m and (B) 1000 m of study sites in urban green spaces. City names and populations are given on the *x*‐axes. Please note the logarithmic scale for the *y*‐axes.

Population‐weighted *Borrelia* infection risk estimates varied between 0 and 7 × 10^4^, indicating high spatial variation in risk—even within individual cities (Figure 5 and Table S4). We did not observe correlation between the total population of the city and the maximum numbers of people estimated to live 500–1000 m from the studied urban green spaces [for 500 m: *r*(35) = 0.07, *p* = 0.68; for 1000 m: *r*(35) = 0.05, *p* = 0.76] (Table S4). However, there were positive correlations between population density–weighted infection risk and the total population of the city [for 500 m: *r*(35) = 0.44, *p* = 0.006; for 1000 m: *r*(35) = 0.43, *p* = 0.007].

## 4 Discussion

Ticks and TBPs were common and widespread in urban green spaces in Finland, indicating that there is a constant and tangible risk of obtaining TBDs within cities. These observations conform to findings from previous studies (Laaksonen et al. 2017; Sormunen et al. 2023), as well to the ongoing nationwide crowdsourcing campaign (www.punkkilive.fi/en), which has indicated that high numbers of tick contacts occur within cities in Finland. However, spatial heterogeneity in tick abundance was also observed to be high across urban green spaces, with implications for the localised risk of TBDs (Estrada‐Peña 2003; Millins et al. 2016; Cat et al. 2017). The main significance of urban green spaces regarding TBDs is that human activity is at its highest therein, which may lead to higher rates of contacts with infected ticks (Hansford et al. 2022; Sormunen, Kulha, et al. 2020; Janzén et al. 2024; Dumas et al. 2024). Correspondingly, it has been observed that the risk of TBDs may be high in urban green spaces, potentially even higher than in more natural areas, despite typically lower hazard levels (Sormunen, Kulha, et al. 2020; Janzén et al. 2024; Hassett et al. 2022). In addition, urban green spaces are also commonly spatially restricted, forming enticing targets for TBD risk control campaigns. Indeed, reducing tick and TBP occurrence in urban green spaces might offer an efficient strategy to prevent TBD cases.

We observed *Borrelia*‐infected *I. ricinus* nymphs from May to September, with the highest prevalence in August and September, whereas infected adult *I. persulcatus* were observed only from May to July. It has previously been observed that tick activity explains most of the variance in tick entomological risk indices regarding *Borrelia* (Sormunen, Kulha, et al. 2020). The lack of infected *I. persulcatus* in August and September observed here was most likely solely due to no or very few actively questing ticks present during these months, rather than any temporal variation in *Borrelia* prevalence. These results conform to previous reports regarding the activity periods of both these species in Finland (Sormunen, Kulha, et al. 2020; Sormunen, Andersson, et al. 2020; Pakanen et al. 2020; Cayol et al. 2017), as well as more general trends observed in European cities regarding *I. ricinus* (Hansford et al. 2022). Consequently, the risk period for TBDs is much shorter in areas of *I. persulcatus* occurrence, and the numbers of locally acquired TBDs may thus be influenced by the tick species present. We also produced population density–weighted risk indices for our study sites and observed high variation in values, indicating differences in local entomological risk indices and/or the population at risk. Interestingly, we observed no obvious correlation between the total population of a city and the observed maximum population densities adjacent to urban green spaces, indicating that even smaller cities may have similar numbers of inhabitants proximate to green spaces as larger ones—in other words, individual green spaces in smaller cities may present a similar TBD hazard as those in larger cities. However, a slight positive correlation between total population and the measured weighted risk indices was also observed, suggesting that risk is generally, and perhaps expectedly, higher in larger cities. Finally, it should be highlighted that we screened for *Borrelia* on the genus level, so positive samples included in our risk assessments may also include non‐pathogenic genospecies. However, species other than pathogenic *Borrelia afzelii, Borrelia garinii* and *B. burgdorferi* s.s. are relatively rare in Finland (Sormunen, Kulha, et al. 2020; Sormunen, Andersson, et al. 2020; Klemola et al. 2019), so the impact on the risk indices is expected to be limited.

We also aimed to identify landscape features that influence *I. ricinus* densities across all the studied cities. We observed positive associations with the percentage of forest cover adjacent to study sites, but negative associations with forest size for both nymphs and adults. Furthermore, nymph densities appeared to be highest when artificial surfaces covered slightly over half of the available area, with lower densities observed when proportions were lower or higher. All these observations could possibly be explained by considering the presence and movements of host animals within urban green spaces. While the densities of many host animals may be equivalent or even higher in urban green spaces than in rural areas (Gallo et al. 2017), artificial surfaces in cities form impassable obstacles and/or funnel movements to specific areas within the urban green space network, restricting the movement opportunities of host animals (Ditchkoff, Saalfeld, and Gibson 2006). As such, hosts may frequent the same plots of land more commonly and/or obtain higher local densities due to restricted dispersal of juveniles, leading to higher host‐finding rates, survival and reproductive success for ticks (within the context of the urban environment—tick densities tend to generally be higher in more natural areas; Bourdin et al. 2023). In conclusion, many host species, particularly medium‐ and large‐sized mammals, require forests to thrive within cities (positive association with forest cover), but when the forest is sufficiently large, the clustering effect on host animals may be lost (negative association with increasing forest size). For artificial surfaces, too much artificial surface may mean that there is insufficient forest or other natural habitats to support host/tick populations, whereas too little artificial surface means a higher proportion of habitats suitable for hosts/ticks and less host clustering. Finally, it should be noted that forests of different sizes within the city may additionally/alternatively serve as sources and sinks—populations of medium and large host animals may mostly inhabit larger forest areas and only occasionally visit smaller forested sites. During visits, ticks drop off from the hosts, but since the larger hosts are not present in these sites constantly, higher numbers of nymphal and adult ticks remain on‐site without blood meals and can be found by cloth dragging.

In any case, it is worth noting that results from individual cities are likely not applicable universally. Indeed, it is likely that urban green spaces do not form a uniform ‘habitat type’ to which specific attributes and corresponding suitability for ticks or TBPs can easily be applied. Apart from being areas with different flora and fauna, green spaces in cities are also influenced by the structure and organisation of the city, and are intrinsically linked to the environments, climate, accessibility and topography of their surroundings. Cities may also influence their own local climates—for example, the strength of the urban heat island (UHI) effect (meaning that cities are often warmer than surrounding areas) is dependent on the size (population) of the city (Zhou, Rybski, and Kropp 2017). Furthermore, the geographical location and spatial context of a city, such as topography and proximity of large water bodies, may influence the warming caused by UHI (Hjort, Suomi, and Käyhkö 2016), possibly even reversing the effect in high‐latitude coastal cities (Suomi and Käyhkö 2012). As such, the effect of such phenomena on tick occurrence may be vastly different between cities and, particularly, between countries (Kulha et al. 2022). In addition, the exact areas in the city where ticks occur and prosper are likely highly influenced by the presence, abundance and movements of host animals (Rizzoli et al. 2014; Hansford et al. 2022). Unfortunately, the environmental variables commonly used to study tick occurrence, including those used here, are too coarse to encompass host‐specific preferences at the relevant scale. Likewise, host data itself, at a sufficient resolution, is nearly non‐existent. Finally, the relative importance of a single host species may likewise depend on several factors, including the suitability of the habitat and the availability of other hosts (Sormunen et al. 2024). The production of more accurate and representative data on host animals and their movement patterns in urban green spaces could provide more accurate and relevant data than landscape features for identifying tick risk areas within cities.

*Borrelia* species were observed from nearly all urban study sites, indicating that infections may be obtained practically anywhere, even within cities (within the confines of ‘green space’). The widespread presence of *Borrelia* spp. in urban green spaces has previously been documented Europe‐wide (Hansford et al. 2022), with prevalence rates commonly comparable to those in more natural environments (Strnad et al. 2017; Estrada‐Peña et al. 2018). In general, the prevalence rates observed in cities in Finland were higher than the reported Europe‐wide mean (Hansford et al. 2022). This data indicates that conditions in urban green spaces are commonly suitable for the circulation of *Borrelia* spp., even if some host animals perceived as important reservoirs, such as voles, may be present in lesser numbers in these highly built‐up environments (Sormunen et al. 2024). Red squirrels ( *Sciurus vulgaris* ) have recently been observed to have an enhanced role in the upkeep of ticks and *Borrelia* in urban green spaces in the largest city of Finland, Helsinki, compared to more sylvatic areas, forming potential targets for risk mitigation campaigns (Sormunen et al. 2024). The role of these synanthropic animals in tick and TBP upkeep particularly in urban green spaces needs to be further elucidated.

*Rickettsia* species were also detected in every city and nearly all the urban study sites. Overall, the prevalence of *Rickettsia* spp. in *I. ricinus* nymphs in urban green spaces in Finland appears to be higher than what has been reported from more rural or less urbanised areas of the country (Sormunen et al. 2016, 2018; Sormunen, Kulha, et al. 2020; Sormunen, Andersson, et al. 2020). The specific factors contributing to these beneficial conditions for the circulation of the bacteria in highly urbanised environments remain undetermined, but studies have suggested a role for European hedgehogs ( *Erinaceus europaeus* ) in the upkeep of *Rickettsia* spp. (Jahfari et al. 2017; Speck et al. 2013; Stanczak et al. 2016). The density of hedgehogs is often higher in urban and suburban areas than in forested or rural areas (Harris and Yalden 2008). Other such synanthropic animals present in Finland are common rats ( *Rattus norvegicus* ) and red squirrels, of which at least the latter have also been suggested to be involved in the circulation of *Rickettsia* spp. (Lipatova, Vitaitė, and Paulauskas 2017). Finally, the potential role of domestic dogs in the upkeep of urban tick populations and TBP circulation remains unknown. However, several species of *Rickettsia* have been shown to be transovarially transmitted (Hauck, Jordan, et al. 2020). An unstudied possibility is that the lower *Rickettsia* spp. prevalence in more natural areas in Finland is maintained mostly by transovarial transmission, whereas higher densities of potential synanthropic reservoir hosts—or less diversity in possible hosts—in urban green spaces provide more opportunities for additional horizontal transmission, leading to higher prevalence.

## 5 Conclusions

Ticks and TBPs are very common in urban green spaces in Finland. Although highly built‐up areas appear suboptimal for the circulation of some TBPs, enzootic cycles for *Borrelia* and *Rickettsia* species appear to be well maintained even within cities. Consequently, there is widespread risk of contacts with ticks carrying *Borrelia* therein. To assess the risk these pathogens pose in urban settings, the incorporation of human density or activity data into entomologic hazard indices should be further explored. Here, we show that population density–weighted risk can vary widely even within an individual city, but also that green spaces of roughly similar risk levels can be found in cities of different sizes. As for factors that determine the quality of habitats for tick and TBP upkeep within urban green spaces, more data are required. Our results seem to indicate that nymph densities are higher in medium‐sized urban forests than in small or large urban forests, but the exact reasons for this remain unknown. In any case, broad generalisations regarding important predictors based on single or few cities—or a single study year—should be avoided. Human activity is at its highest in urban green spaces, so any efforts to reduce tick risk therein may lead to tangible reductions in TBDs and are thus worthy of pursuing.

## Ethics Statement

An ethics statement is not applicable as this study was not classified as a human or animal experiment. Tick samples utilised in this study were field‐collected from non‐protected areas.

## Conflicts of Interest

The authors declare no conflicts of interest.

## Supporting information


Appendix S1

## Data Availability

The data that support the findings of this study are available from the corresponding author upon reasonable request.
