# Supplementary material for: There Goes the Neighbourhood—A Multi‐City Study Reveals Ticks and Tick‐Borne Pathogens Commonly Occupy Urban Green Spaces
Source: Zoonoses Public Health. 2025 Jan 16;72(3):313–23. doi: 10.1111/zph.13208 (PMC11967321; doi:10.1111/zph.13208)
Supplement: Supplementary file 1 — Appendix S1 [file ZPH-72-313-s001.docx]

**There goes the neighbourhood - A multi-city study reveals ticks and tick-borne pathogens commonly occupy urban green spaces**

Jani J. Sormunen, Satu Kylänpää, Ella Sippola, Riikka Elo, Nosheen Kiran, Veli-Matti Pakanen, Eva R. Kallio, Eero J. Vesterinen, Tero Klemola

TECHNICAL APPENDIX

[**Table S1. Study sites, numbers of tick caught and distances dragged** 1](#_Toc179661992)

[**Text S1: Additional information on real-time quantitative PCR** 3](#_Toc179661993)

[**Table S2. List of primers and probes used for detection of tick-borne pathogens** 4](#_Toc179661994)

[**Table S3. Mastermix contents for qPCR screening of pathogens** 5](#_Toc179661995)

[**Table S4. Population density weighted infection risks** 6](#_Toc179661996)

[**References** 7](#_Toc179661997)

# **Table S1. Study sites, numbers of tick caught and distances dragged**

| City |  | Adults/nymphs/larvae (meters dragged) | | | | |
| --- | --- | --- | --- | --- | --- | --- |
|  | Area (id^a^) | May | June | July | August | September |
| Turku | Vasaramäki  (a11) | 12/1/0  (1000) | 3/2/0  (1000) | - | 5/30/6  (500) | 1/5/0  (500) |
| Turku | Luolavuori  (a8) | 5/5/0  (1000) | 1/0/0  (1000) | - | 0/16/0  (500) | 3/14/5  (500) |
| Turku | Virnamäenpuisto  (a9) | 4/3/1  (1000) | 0/3/0  (1000) | - | 3/5/0  (500) | 0/4/1  (500) |
| Turku | Iso-Heikkilä  (a1) | 0/1/0  (1000) | 3/2/0  (1000) | 5/1/0  (1000) | 3/2/0  (400) | 5/26/1  (500) |
| Turku | University Hill  (a6) | 12/19/3  (1000) | 17/26/3  (1000) | - | 18/10/0  (600) | 2/4/39  (500) |
| Turku | Nummi  (a10) | 6/7/0  (1000) | 4/2/0  (1000) | - | 17/44/0  (800) | 2/0/0  (500) |
| Turku | Kasarmialue  (a7) | 4/13/0  (1000) | 22/51/7  (1000) | - | 12/23/0  (600) | 6/9/1  (400) |
| Turku | Kakolanmäki  (a2) | 4/7/0  (1000) | - | 1/0/0  (1000) | 0/5/0  (500) | - |
| Turku | Vartiovuori  (a5) | 2/6/1  (1000) | 0/0/0  (1000) | - | 0/4/0  (500) | 0/1/0  (500) |
| Turku | Samppalinna  (a4) | 5/5/0  (1000) | 2/0/0  (1000) | - | 0/3/1  (500) | 1/0/0  (500) |
| Turku | Urheilupuisto  (a3) | 3/8/0  (1000) | 0/0/0  (1000) | - | 0/3/0 (500) | 0/0/0  (500) |
| Tampere | Pyynikki  (d1) | 24/56/26  (500) | 6/39/82  (500) | 37/41/32  (500) | 38/215/421  (500) | 23/209/95  (500) |
| Tampere | Näsinpuisto  (d2) | 12/22/22  (510) | 5/10/27  (500) | 12/6/2  (500) | 10/117/112  (500) | 9/109/25  (500) |
| Tampere | Kauppi  (d4) | 1/0/0  (500) | 0/0/0  (500) | 0/0/0  (500) | 0/1/0  (500) | 0/1/0  (500) |
| Tampere | Iidesjärvi  (d5) | 0/0/0  (500) | 0/0/0  (500) | 0/0/0  (500) | 1/0/0  (500) | 0/1/0  (500) |
| Tampere | Hatanpää  (d3) | 1/2/0  (500) | 1/0/0  (500) | 7/2/4  (500) | 14/7/254  (500) | 4/6/14  (500) |
| Jyväskylä | Harju  (b5) | 2/0/0  (500) | 1/4/2  (500) | 6/3/23  (500) | 0/1/13  (500) | 6/6/13 (500) |
| Jyväskylä | Hippos  (b1) | 8/9/1  (500) | 6/16/24  (500) | 6/2/0  (500) | - | - |
| Jyväskylä | Ilokivi  (b3) | 5/12/6  (500) | 6/35/215  (500) | 8/9/44  (500) | 1/1/0  (500) | 2/17/12  (500) |
| Jyväskylä | Köyhälampi  (b2) | 1/6/0  (500) | 6/6/28  (500) | 6/4/6  (500) | 2/1/0  (500) | 4/49/0  (500) |
| Jyväskylä | Seminaarimäki  (b4) | 5/14/0  (500) | 3/18/114  (500) | 10/2/5  (500) | 3/0/2  (500) | 18/59/10  (500) |
| Jyväskylä | Tourujoki  (b6) | 1/4/0  (500) | 1/5/1  (500) | 1/0/1  (500) | 5/3/0  (500) | - |
| Jyväskylä | Tuomiojärvi  (b7) | 12/7/1  (500) | 7/30/127  (500) | 14/14/37  (500) | 8/6/57  (500) | 8/55/33  (500) |
| Oulu | Hietasaari  (c5) | 21/13/0  (500) | 12/9/0  (500) | 6/2/0  (500) | 0/5/0  (500) | 0/2/0  (500) |
| Oulu | Kaakkuri  (c2) | 4/3/0  (500) | 1/0/0  (500) | 0/0/0  (500) | 0/0/0  (500) | 0/0/0  (500) |
| Oulu | Kempeleenlahti  (c1) | 37/4/0  (500) | 31/1/0  (500) | 4/2/0  (500) | 0/2/0  (500) | 0/0/0  (500) |
| Oulu | Oritkari  (c3) | 29/11/0  (500) | 25/2/0  (500) | 3/3/0  (500) | 0/0/0  (500) | 1/1/0  (500) |
| Oulu | Pyykösjärvi  (c6) | 14/1/0  (500) | 3/0/0  (500) | 2/1/0  (500) | 1/1/0  (500) | 0/0/0  (500) |
| Oulu | Svaaninsuo  (c4) | 27/14/0  (500) | 7/1/0  (500) | 1/0/0  (500) | 0/0/0  (500) | 0/0/0  (500) |

^a^Id is the panel and number for the specific study site on Figure 1.

# **Text S1: Additional information on real-time quantitative PCR**

The thermal cycling profile used was 95°C for 5 minutes, then 50 cycles of 95°C for 10 sec and 60°C for 30 sec (annealing/extension temperature was 58°C in assays involving *Rickettsia*). For RNA samples, thermal cycling profile was 45°C for 10 minutes (reverse transcription) and 95°C for 2 minutes, then 50 cycles of 95°C for 5 sec and 60°C for 20 sec. Primers and probes used are reported in Table S1, mastermix contents in Table S2. All DNA/RNA samples were analyzed in two replicate reactions carried out on 96-well plates using CFX96 Real-Time Thermal Cycler (BioRad) or QuantStudio 12K Flex Real-Time PCR System (Life Technologies Inc. [LTI], Carlsbad, CA). Samples were considered positive when successful amplification was detected in both replicate reactions or in two consecutive runs.

DNA samples of *Anaplasma phagocytophilum* and *Babesia microti* extracted from the blood of infected voles were used as positive controls for *A. phagocytophilum* and *Babesia* spp. For *Neoehrlichia mikurensis*, we used samples previously found positive and sequenced. For *Rickettsia* and *Borrelia,* we used commercially available controls (*Rickettsia*: ref. MBC042, Vircell, Granada, Spain; *Borrelia*: ref. MBC078-R, Vircell, Granada, Spain). Multiple TBEV RNA strains provided by Anu Jääskeläinen (University of Helsinki) were used as positive controls in TBEV assays. Water was used in negative controls in each assay.

| Primer/probe name | Primer/probe target | 5’ 🡪 3’ | Reference |
| --- | --- | --- | --- |
| qPCR: |  |  |  |
| Bb23Sf | *B. burgdorferi* 23S RNA | CGAGTCTTAAAAGGGCGATTTAGT | Courtney et al. 2004 |
| Bb23Sr | *B. burgdorferi* 23S RNA | GCTTCAGCCTGGCCATAAATAG |  |
| Bb23Sp | *B. burgdorferi* 23S RNA | [FAM]-AGATGTGGTAGACCCGAAGCCGAGTG-[BHQ1] |  |
|  |  |  |  |
| Rspp-F | *Rickettsia gltA* | GAGAGAAAATTATATCCAAATGTTGAT | Labruna et al. 2004 |
| Rspp-R | *Rickettsia gltA* | AGGGTCTTCGTGCATTTCTT |  |
| Rspp-P | *Rickettsia gltA* | [CY5]-CATTGTGCCATCCAGCCTACGGT-[BHQ3] |  |
|  |  |  |  |
| CNeGroEL-F | *N. mikurensis* *groEL* | CCTTGAAAATATAGCAAGATCAGGTAG | Jahfari et al. 2012 |
| CNeGroEL-R | *N. mikurensis* *groEL* | CCACCACGTAACTTATTTAGCACTAAAG |  |
| CNeGroEL-P | *N. mikurensis* *groEL* | [FAM]-CCTCTACTAATTATTGCWGAAGATGTAGAAGGTGAAGC-[BHQ1] |  |
|  |  |  |  |
| ApMSP2F | *A.phagocytophilum Msp2* | ATGGAAGGTAGTGTTGGTTATGGTATT | Courtney et al. 2004 |
| ApMSP2R | *A.phagocytophilum Msp2* | TTGGTCTTGAAGCGCTCGTA |  |
| ApMSP2P | *A.phagocytophilum Msp2* | [CY5]-TGGTGCCAGGGTTGAGCTTGAGATTG-[BBQ650] |  |
|  |  |  |  |
| Bab18S-F | *Babesia* 18S rRNA | CAGCTTGACGGTAGGGTATTGG | Radzijevskaja et al. 2008 |
| Bab18S-R | *Babesia* 18S rRNA | TCGAACCCTAATTCCCCGTTA |  |
| Bab18S-P | *Babesia* 18S rRNA | [HEX]-CGAGGCAGCAACGG-[BHQ1] |  |
|  |  |  |  |
| TBE1-F | TBEV non-struct. prot. 5 | GGGCGGTTCTTGTTCTCC | Schwaiger & Cassinotti 2003 |
| TBE1-R | TBEV non-struct. prot. 5 | ACACATCACCTCCTTGTCAGACT |  |
| TBE1-P | TBEV non-struct. prot. 5 | [FAM]-TGAGCCACCATCACCCAGACACA-[BHQ1] |  |

# **Table S2. List of primers and probes used for detection of tick-borne pathogens**

| qPCR target | Single/  duplex/  multiplex | Sample(s) | Total reaction volume | Probe mix^a^ | ddH2O | Forward/reverse primer concentration | Probe concentration | DNA/RNA sample |
| --- | --- | --- | --- | --- | --- | --- | --- | --- |
| *B. burgdorferi* s.l. | Single | Single | 5 µl | 2.5 µl | 1.25 µl | 200 nM | 100 nM | 1 µl |
| *A. phagocytophilum*  *Babesia* spp.  *N*. *mikurensis* | Multiplex | Pooled | 11 µl | 5.5 µl | 0.75 µl | 400 nM  400 nM  200 nM | 200 nM  200 nM  100 nM | 2 µl |
| *A. phagocytophilum* | Single | Single | 5 µl | 2.5 µl | 1.25 µl | 200 nM | 100 nM | 1 µl |
| *Babesia* spp. | Single | Single | 5 µl | 2.5 µl | 1.25 µl | 200 nM | 100 nM | 1 µl |
| *N mikurensis* | Single | Single | 5 µl | 2.5 µl | 1.25 µl | 200 nM | 100 nM | 1 µl |
| *Rickettsia* spp. | Single | Pooled | 8 µl | 4 µl | 1.13 µl | 300 nM | 150 nM | 1.4 µl |
| *Rickettsia* spp. | Single | Single | 5 µl | 2.5 µl | 1.25 µl | 200 nM | 100 nM | 1 µl |
| TBEV | Single | Pooled | 8 µl | 4 µl | 0.13 µl | 400 nM | 100 nM | 3 µl |
| TBEV | Single | Single | 5 µl | 2.5 µl | - | 400 nM | 100 nM | 1.9 µl |

# **Table S3. Mastermix contents for qPCR screening of pathogens**

^a^DNA samples: SensiFAST™ Probe Lo-ROX Kit; For RNA samples: SensiFAST™ Probe Lo-ROX One-Step Kit

| Study site | City^a^ | Population density | | DIN/DIA^b^ | Weighted infection risk | |
| --- | --- | --- | --- | --- | --- | --- |
|  |  | 500 m | 1000 m |  | 500 m | 1000 m |
| Näsinpuisto | Tampere | 22390 | 37949 | 0.98 | 2.2 x 10^^4^ | 3.7 x 10^^4^ |
| Pyynikki | Tampere | 9610 | 21109 | 1.3 | 1.2 x 10^^4^ | 2.6 x 10^^4^ |
| Kauppi | Tampere | 2696 | 14118 | 0 | 0 | 0 |
| Iidesjärvi | Tampere | 7826 | 25239 | 0 | 0 | 0 |
| Hatanpää | Tampere | 20997 | 24611 | 0.13 | 2.6 x 10^^3^ | 3.1 x 10^^3^ |
| Svaaninsuo | Oulu | 7650 | 12053 | 0.65 | 5 x 10^^3^ | 7.8 x 10^^3^ |
| Kaakkuri | Oulu | 4307 | 5345 | 0.12 | 5 x 10^^2^ | 6.4 x 10^^2^ |
| Hietasaari | Oulu | 52 | 2434 | 0.72 | 37 | 1.7 x 10^^3^ |
| Kempeleenlahti | Oulu | 1034 | 1340 | 1.9 | 1.9 x 10^^3^ | 2.5 x 10^^3^ |
| Oritkari | Oulu | 0 | 6452 | 1 | 0 | 6.6 x 10^^3^ |
| Pyykösjärvi | Oulu | 1344 | 4507 | 0.12 | 1.6 x 10^^2^ | 5.4 x 10^^2^ |
| Harju | Jyväskylä | 17907 | 32467 | 0.21 | 3.8 x 10^^3^ | 6.9 x 10^^3^ |
| Hippos | Jyväskylä | 11447 | 25275 | 0 | 0 | 0 |
| Ilokivi | Jyväskylä | 11162 | 26318 | 0.75 | 8.4 x 10^^3^ | 2 x 10^^4^ |
| Köyhälampi | Jyväskylä | 6974 | 15148 | 0.45 | 3.2 x 10^^3^ | 6.9 x 10^^3^ |
| Seminaarimäki | Jyväskylä | 11162 | 27891 | 0.83 | 9.3 x 10^^3^ | 2.3 x 10^^4^ |
| Tourujoki | Jyväskylä | 13838 | 26729 | 0.25 | 3.5 x 10^^3^ | 6.7 x 10^^3^ |
| Tuomiojärvi | Jyväskylä | 13508 | 18179 | 0.95 | 1.3 x 10^^4^ | 1.7 x 10^^4^ |
| Ylipistonmäki | Turku | 14379 | 44614 | 0.31 | 4.5 x 10^^3^ | 1.4 x 10^^4^ |
| Kasarmialue | Turku | 17090 | 38250 | 0.8 | 1.4 x 10^^4^ | 3.1 x 10^^4^ |
| Vasaramäki | Turku | 5764 | 14258 | 0.23 | 1.3 x 10^^3^ | 3.2 x 10^^3^ |
| Luolavuori | Turku | 6433 | 22617 | 0.29 | 1.9 x 10^^3^ | 6.6 x 10^^3^ |
| Vartiovuori | Turku | 29457 | 42965 | 0.05 | 1.4 x 10^^3^ | 2.1 x 10^^3^ |
| Samppalinna | Turku | 23050 | 55443 | 0.03 | 7.5 x 10^^2^ | 1.8 x 10^^3^ |
| Urheilupuisto | Turku | 24713 | 47654 | 0.03 | 7.1 x 10^^2^ | 1.4 x 10^^3^ |
| Virnamäki | Turku | 10745 | 17102 | 0.06 | 5.9 x 10^^2^ | 9.4 x 10^^2^ |
| Nummi | Turku | 6869 | 17542 | 0.38 | 2.6 x 10^^3^ | 6.6 x 10^^3^ |
| Kakolanmäki | Turku | 19967 | 34167 | 0 | 0 | 0 |
| Iso-Heikkilä | Turku | 4533 | 17678 | 0.05 | 2.3 x 10^^2^ | 8.8 x 10^^2^ |
| Hakuninmaa | Helsinki | 5846 | 12991 | 0.37 | 2.2 x 10^^3^ | 4.8 x 10^^3^ |
| Lapinniemi | Helsinki | 22961 | 54330 | 0.24 | 5.5 x 10^^3^ | 1.3 x 10^^4^ |
| Kumpula | Helsinki | 18127 | 31262 | 2.2 | 4 x 10^^4^ | 7 x 10^^4^ |
| Laakso | Helsinki | 22495 | 42958 | 1.1 | 2.5 x 10^^4^ | 4.7 x 10^^4^ |
| Lauttasaari | Helsinki | 10699 | 21013 | 0.81 | 8.7 x 10^^3^ | 1.7 x 10^^4^ |
| Lehtisaari | Helsinki | 1977 | 6502 | 0.63 | 1.3 x 10^^3^ | 4.2 x 10^^3^ |
| Meilahti | Helsinki | 21469 | 31793 | 1.28 | 2.7 x 10^^4^ | 4.1 x 10^^4^ |
| Seurasaari | Helsinki | 10 | 5962 | 0.36 | 4 | 2.1 x 10^^3^ |

# **Table S4. Population density weighted infection risks**

^a^City populations: Tampere = 249 000; Oulu = 212 000; Jyväskylä = 146 000; Turku = 198 000; Helsinki = 665 000.

^b^Study site specific density of nymphs (Tampere, Jyväskylä, Turku, Helsinki) or adults (Oulu) infected with *Borrelia* spp.

# **References**

[1] J. W. Courtney, L. M. Kostelnik, N. S. Zeidner, and R. F. Massung, "Multiplex Real-Time PCR for Detection of *Anaplasma phagocytophilum* and *Borrelia burgdorferi*," *Journal of Clinical Microbiology,* vol. 42, no. 7, pp. 3164-3168, 2004, doi: 10.1128/JCM.42.7.3164-3168.2004.

[2] M. Vayssier-Taussat, S. Moutailler, L. Michelet, E. Devillers, S. Bonnet, and J. Cheval, "Next generation sequencing uncovers unexpected bacterial pathogens in ticks in Western Europe," *PLoS One,* vol. 8, 2013// 2013, doi: 10.1371/journal.pone.0081439.

[3] M. B. Labruna, T. Whitworth, M. C. Horta, D. H. Bouyer, J. W. McBride, and A. Pinter, "*Rickettsia* species infecting *Amblyomma cooperi* ticks from an area in the State of São Paulo, Brazil, where Brazilian spotted fever is endemic," *J Clin Microbiol,* vol. 42, 2004// 2004, doi: 10.1128/JCM.42.1.90-98.2004.

[4] S. Jahfari *et al.*, "Prevalence of *Neoehrlichia mikurensis* in ticks and rodents from North-west Europe," *Parasites & Vectors,* vol. 5, no. 1, p. 74, 2012/04/19 2012, doi: 10.1186/1756-3305-5-74.

[5] J. Radzijevskaja, A. Paulauskas, and O. Rosef, "Prevalence of *Anaplasma phagocytophilum* and *Babesia divergens* in *Ixodes ricinus* ticks from Lithuania and Norway," *International Journal of Medical Microbiology,* vol. 298, pp. 218-221, 2008/09/01/ 2008, doi: <https://doi.org/10.1016/j.ijmm.2008.01.008>.

[6] M. Schwaiger and P. Cassinotti, "Development of a quantitative real-time RT-PCR assay with internal control for the laboratory detection of tick borne encephalitis virus (TBEV) RNA," *Journal of Clinical Virology,* vol. 27, no. 2, pp. 136-145, 2003/07/01/ 2003, doi: <https://doi.org/10.1016/S1386-6532(02)00168-3>.
